# A study of the transformation of umbilical cord mesenchymal stem cells by interferon-gamma 

**DOI:** 10.22038/ijbms.2021.56619.12639

**Published:** 2021-09

**Authors:** Kalina Belemezova, Ivan Bochev, Ekaterina Ivanova-Todorova, Stanimir Kyurkchiev, Dobroslav Kyurkchiev

**Affiliations:** 1 Laboratory of Clinical Immunology, University Hospital St. Ivan Rilski, Department of Clinical Immunology, Medical Faculty, Medical University of Sofia, Sofia, Bulgaria, Ob/Gyn Hospital Dr Shterev, Sofia, Bulgaria; 2 Department of Molecular Immunology, Institute of Biology and Immunology of Reproduction, Bulgarian Academy of Sciences, Sofia, Bulgaria, Ob/Gyn Hospital Dr Shterev, Sofia, Bulgaria; 3 Laboratory of Clinical Immunology, University Hospital St. Ivan Rilski, Department of Clinical Immunology, Medical Faculty, Medical University of Sofia, Sofia, Bulgaria; 4 Ob/Gyn Hospital Dr Shterev, Sofia, Bulgaria

**Keywords:** Cytokine, Differentiation, Inflammation, PD-L1, Proliferation, Stem cells

## Abstract

**Objective(s):**

Mesenchymal stem cells (MSCs) exist in almost all tissues. Their unique nature is completed by their immunomodulatory functions, holding promise for the treatment of many diseases. An inflammatory environment precedes the immunosuppressive abilities of MSCs and this study was intended to better understand how umbilical cord MSCs (UCMSCs) react to the process of inflammation, regarding their basic characteristics and behavior when primed with the key pro-inflammatory cytokine, Interferon-γ (IFNγ).

**Materials and Methods:**

Human MSCs from the umbilical cord were isolated, expanded, and treated with IFNγ. Primed cells were analyzed to define their ability to form colonies, their morphology, differentiation potential, proliferation, and apoptosis rate.

**Results:**

UCMSCs treated with IFNγ changed their fibroblast-like morphology and retained the expression of typical MSCs markers. IFNγ treated UCMSCs had significantly higher MFI levels regarding the expression of HLA-I (980.43 ± 556.64) and PD-L1 (598.04 ± 416.90) compared with the control cells (144.97 ± 78.5 and 122.05 ± 103.83, respectively; *P*<0.01). Under the influence of IFNγ, the cells had a lower population doubling time compared with the control cultures (50.345 ± 9.155 versus 61.135 ± 21.110, respectively; *P*<0.01) and higher numbers of colony-forming unit-fibroblasts (26.0 ± 12.2 versus 10.2 ± 8.0, respectively; *P*<0.05). The primed MSCs could not undergo osteogenic and adipogenic differentiation. IFNγ increased the percentage of cells in the apoptotic state on day eight (29.470 ± 6.59 versus 15.708 ± 6.190, respectively; *P*<0.01).

**Conclusion:**

The properties of UCMSCs can be influenced by the pro-inflammatory cytokine IFNγ.

## Introduction

Mesenchymal stem cells (MSCs) have been of great interest among scientists in recent years for several reasons. They have a potential for self-renewal and can be obtained from multiple sources like bone marrow, adipose tissue, Wharton’s jelly, skin, muscle, and dental pulp. MSCs are plastic-adherent with a fibroblast-like shape and they present a specific phenotype by the expression of certain surface molecules like CD105, CD73, CD90, CD44, and CD29, but lack any hematopoietic markers (1). They also have the *in vitro* capacity to differentiate into osteoblasts, adipocytes, and chondroblasts (2, 3). Because of their properties and involvement in many physiological and pathological processes, including tissue damage and regeneration, inflammatory diseases, and aging, MSCs have encouraged scientists to seek suitable ways to use these cells to treat various diseases (4-7). 

Perhaps the most intriguing aspect of MSCs’ nature, which is still not fully understood, is their immunomodulatory functions. MSCs modulate the functions of various immune cells, including T and B cells, dendritic cells, and natural killer cells (8). Although MSCs are said to be “immune-privileged”, they do respond to inflammation. The inflammatory environment exists during any tissue damage and MSCs are exposed to its stimuli during various clinical conditions. In an inflammatory environment with high levels of pro-inflammatory cytokines (IFNγ, TNFα, and IL-1β), MSCs are activated and adopt an immunosuppressive phenotype. Several studies indicate that priming by inflammatory cytokines is essential for MSC-mediated immunosuppression (9-11). 

The pro-inflammatory cytokine interferon-γ (IFNγ) has been the most extensively used cytokine for priming MSCs. Indeed, the International Society for Cellular Therapy (ISCT) recommends it as a standard priming method for evaluating the immunosuppressive capacity of MSCs *in vitro* (12). IFNγ is a potent pro-inflammatory cytokine that is produced by multiple cell types including activated T cells, NK cells, NKT cells, and macrophages, and plays important and complex roles in both innate and adaptive immune responses. 

In the future, MSCs are expected to be used as a therapeutic tool for treating injuries and trauma of the nervous system, cardiovascular diseases, bone defects and fractures, osteoarthritis, and cartilage damage, to repair large skin defects such as wounds and scars, for autoimmune diseases, as well as in cosmetic medicine. Understanding the interaction between MSCs and the environment in which they are located during inflammation gives us important information about the *in vivo* mechanisms involved in MSC-mediated therapeutic effects and undoubtedly contributes to the development of protocols for clinical application of MSCs.

## Materials and Methods

***UCMSCs isolation and culture***

Human umbilical cord mesenchymal stem cells (UCMSCs) were isolated from healthy donors (gestational age 37–40 weeks) after obtaining a signed written informed consent approved by the Ethics Committee of Ob/Gyn Hospital Dr Shterev, Sofia, Bulgaria. Umbilical cords’ pieces (about 20 cm in length) were briefly washed with 70% ethanol and then stored in a sterile saline solution. Blood vessels were carefully removed and the rest of the tissue was cut into small pieces, which were then incubated in collagenase I (0.25%, Genaxxon, Germany), hyaluronidase (40 mg/ml, Genaxxon, Germany), and Penicillin/Streptomycin/Amphotericin B Mix (1%, PAN Biotech, Germany) at 37°C in a humidified, 5% CO_2_ incubator, on a shaker, for two hours. Next, the homogenized tissue was diluted with sterile saline solution and filtered through a 70 µm cell strainer (Greiner bio-one, Austria). Samples were centrifuged for 10 min at 300 x g and cell pellets were seeded in 25 cm^2^ cell culture flasks (EuroClone, Italy) at a density of 1x104/cm^2^ in DMEM/F12 (PAN Biotech, Germany) supplemented with fetal bovine serum (10%, FBS, PAN Biotech, Germany), rHuFGF-b (10 ng/ml, Genaxxon, Germany) and Penicillin/Streptomycin/Amphotericin B Mix. Cell cultures were maintained in a humidified, 5% CO2 incubator at 37°C. When cultures reached 80-90 % confluence, cells were harvested (Trypsin 0,05% / EDTA 0.02%; PAN Biotech, Germany), counted and expanded. UCMSCs between second and fourth passages were used for the subsequent analyses (13).

***MSCs priming***

UCMSCs were treated with recombinant IFNγ (Roche, Switzerland) at a concentration of 100 IU/ml. The concentration of IFNγ used was chosen based on published reports (14, 15) and our own preliminary results (data not shown). Primed cells were cultured in DMEM/F12 supplemented with FBS (10%) and Penicillin/Streptomycin/Amphotericin B Mix (1%). Cell culture medium was changed every 48-72 hr and fresh IFNγ was added. The period of IFNγ treatment depended on the type of experiment performed. 

***UCMSCs colony formation assay***

One of the key characteristics of MSCs is their ability to form colonies derived from a single cell when seeded at low concentrations *in vitro*. The precursor cells possessing colony-forming ability are known as colony-forming unit-fibroblasts (CFU-F). UCMSCs at passage two were seeded in 25 cm^2^ cell culture flasks at a concentration of 20 cell/cm^2^ and were maintained in DMEM/F12 supplemented with FBS (10%), Penicillin/Streptomycin/Amphotericin B Mix (1%), and IFNγ (100 IU/ml). The cell culture medium was changed every 48–72 hr and fresh IFNγ was added to the cell cultures. Control UCMSCs were cultured in a standard culture medium without the addition of IFNγ, as described above. Using an inverted microscope (Leica Microsystems) the formation of colonies was assessed daily. After 14 days the colony-forming units were treated with fixating/staining solution containing crystal violet solution (0.05%), formaldehyde (1%), phosphate-buffered saline (10%), and methanol (10%) for 20 min at RT. Afterward, the excess dye was washed several times briefly. Colonies formed by CFU-F consisting of at least 50 cells were counted on a stereomicroscope. 

***Flow cytometry***

UCMSCs at passage two were seeded at a concentration of 1x10^5^ cells/cm^2^ and cultured in the presence of IFNγ for five days. Control cells were kept in a medium without IFNγ. UCMSCs were trypsinized, counted, and 1x10^6^ cells/sample were centrifuged for 10 min at 300 x g. Cells were washed (CellWash solution, Becton Dickinson, USA) and labeled with fluorochrome-conjugated antibodies for 15 min in the dark. After labeling, cells were washed again and fixed with FIX solution (BD, USA). Cell surface markers of UCMSCs were analyzed using the following antibodies: anti-CD45-FITC/CD34-PE, -CD73-PE, -CD90-FITC, -CD105-PerCP/Cy5-5, -CD44-FITC, -CD29-PE, -CD146-PE, -HLA-I-FITC, -PD-L1-PE (all from eBioscience, USA). Non-specific background fluorescence was determined using appropriate isotype controls. The specific fluorescent labeling was analyzed on a FACSCalibur flow cytometer (BD, USA) using the BD CellQuest Pro software (BD, USA).

***UCMSCs proliferation assay and population doubling time (PDT)***

The AlamarBlue (AB) assay (Bio-Rad Laboratories) is an indirect measure of cell numbers and produces linear results with high specificity and sensitivity. AlamarBlue assay was conducted to determine cell viability and proliferation rate. UCMSCs at passage two were trypsinized and seeded in 96-well plates at a concentration of 500 cells/well, in quadruplets. Cells were exposed to IFNγ throughout the experiment, while control cells were kept only in a standard culture medium. Cell proliferation was measured every day for a period of 7 days using AlamarBlue, according to the manufacturer’s instructions. Briefly, UCMSCs were incubated for 4 hr at 37 °C in a humidified, 5% CO_2_ incubator with 1/10^th^ volume Alamar Blue in the culture medium. Afterward, fluorescence was measured at wavelengths of 544 nm (excitation) and 590 nm (emission) on a FLUOstar OPTIMA microplate reader (BMG Labtech). Cell proliferation rate and PDT in each experimental condition were calculated using a standard curve built from measuring the AB fluorescence intensity of control untreated cells seeded at well-known densities. 

The population doubling time (PDT) was calculated using the formula:

*PDT = (t – t*_0_ *) x log2/log (C – C*_0_ *)*,

where t-t_0_ is culture time (h); C is the final cell concentration; C_0_ is the initial cell concentration.

***Osteogenic differentiation***

UCMSCs were plated at a density of 5x10^4 ^cell/cm^2^ and cultured in a standard culture medium. After reaching 80% confluence, an osteogenic differentiation-inducing medium consisting of DMEM/F12 supplemented with FBS (10%), Penicillin/Streptomycin/Amphotericin B Mix (1%), dexamethasone (100 nmol/l, Sigma-Aldrich, USA), ascorbic acid (0.2 mmol/l, Sigma-Aldrich, USA), b-glycerophosphate (10 mmol/l, Sigma-Aldrich, USA) and IFNγ (100 IU/ml) was added (16). The osteogenic induction medium was replaced every 48-72 hr for 21 days. For the positive control, cells were maintained in an osteogenic inducing medium lacking IFNγ and for the negative control, cells were cultured in a regular medium (DMEM/F12/FBS/Penicillin/Streptomycin/

Amphotericin B Mix). Successful differentiation was determined using Alizarin red S staining, Von Kossa staining method, and alkaline phosphatase (ALP) activity measurement. 

***Alizarin red S staining***

Alizarin red S staining was used to locate calcium deposits in cell cultures. Briefly, cell cultures were washed with saline solution and fixed with paraformaldehyde (4%) for 30 min at RT. Fixed cells were then incubated with Alizarin red S (2%, Sigma-Aldrich) solution at pH 4.2 for 40 min at RT. Finally, the excess stain was washed with distilled water.

***Von Kossa staining assay***

UCMSCs treated with osteogenic inducing medium with or without IFNγ and control cells were washed with distilled water and incubated with silver nitrate (1% w/v) solution for one hour under ultraviolet light (λ = 366 nm). Under these conditions, the calcium deposits are stained black. Afterward, the excess silver nitrate solution was washed off. 

***Alkaline phosphatase assay***

UCMSCs were washed with phosphate-buffered saline pH 7.2–7.4 and then permeabilized with Triton X-100 (0.2%, Sigma-Aldrich) in alkaline phosphatase buffer (Na_2_CO_3 _0.05 mol/l, MgCl_2 _0.5 mmol/l pH 9.5) for 20 min. As a substrate for the alkaline phosphatase 4-p-nitrophenyl phosphate (pNPP; Sigma-Aldrich) was used. The substrate (pNPP 3.5 mmol/l in ALP buffer) was added to the cell lysates and incubated for 20 min at RT. Finally, the absorbance of the colored solution was measured spectrophotometrically at 405 nm wavelength (FLUOstar OPTIMA, BMG Labtech).

***Adipogenic differentiation***

In order to induce adipogenic differentiation, UCMSCs were plated at a density of 5x10^4^ cell/cm^2^ and cultured in a standard culture medium (17). After reaching 80% confluence adipogenic differentiation-inducing medium consisting of DMEM/F12 supplemented with DMEM/F12/FBS/penicillin/streptomycin/amphotericin B Mix, dexamethasone (1 µmol/l, Sigma-Aldrich, USA), bovine insulin (10 mg/mL, Sigma-Aldrich, USA), 3-isobutyl-1-methyl-xanthine (0.5 mmol/l, IBMX; Sigma-Aldrich, USA), and indomethacin (60 mmol/l, Sigma-Aldrich, USA) was added. During the differentiation period, primed cells were kept in a differentiation-inducing medium supplemented with IFNγ (100 IU/ml). The positive control cells were maintained in an adipogenic-inducing medium lacking IFNγ and for the negative control, cells were cultured in a regular medium (DMEM/F12/FBS/ penicillin/streptomycin/amphotericin B Mix). The cell culture medium was replaced every 48-72 hr for 21 days. Successful differentiation was confirmed with Oil Red O (Sigma-Aldrich, USA) staining of formed lipid vacuoles. 

***Oil Red O staining***

Cell cultures were washed with saline solution and fixed with paraformaldehyde 4% for 30 min at RT. The fixed cells were washed with distilled water and stained with freshly prepared Oil Red O solution 0.6% for one hour at room temperature. Afterward, the excess stain was washed away and the presence or absence of stained in red lipid drops was observed through an inverted light microscope (Leica, Germany).

***UCMSCs apoptotic assay***

UCMSCs at passage two were seeded at a concentration of 1x10^5^ cells/cm^2^ and cultured in DMEM/F12 supplemented with FBS (10%) and antibiotic/antimycotic (1%) in the presence or absence of IFNγ for eight days (18). Cells were harvested at days one, four, and eight, and the percentage of cells in early (FITC Annexin V positive and propidium iodide (PI) negative) and late apoptosis (both FITC Annexin V and PI positive) was determined using FITC Annexin V Apoptosis Detection Kit I (BD, USA) according to manufacturer’s instructions. The samples were analyzed on a FACSCalibur flow cytometer using the BD CellQuest Pro software.

***Statistical analysis***

Statistical analysis was performed using SigmaPlot (v12.5, Systat Software, Inc.) statistical software. All data are presented as means with standard deviation (± SD). Where appropriate, Student’s paired t-test, one-way repeated-measures ANOVA, or non-parametric Wilcoxon signed-rank test for related data were performed. For all analyses, a level of *P*≤0.05 was considered statistically significant. Regression analyses were performed using CurveExpert Basic (v1.4, Hyams Development) statistical software.

## Results

***Primed UCMSCs morphology***

**Figure 1:**
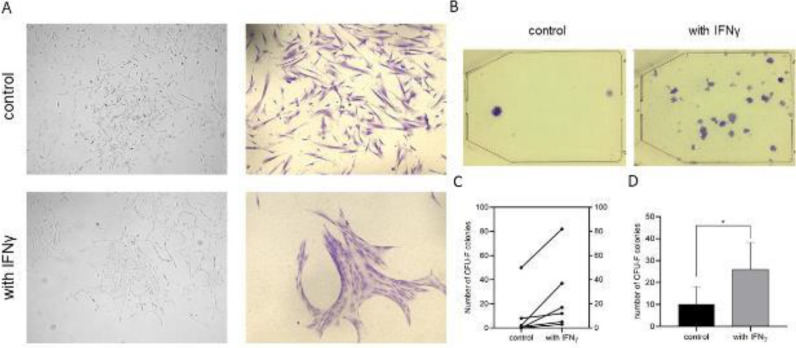
Morphology and clonogenicity of control and IFNγ treated UCMSCs

Following the addition of IFNγ to the cell culture medium, changes in the cell morphology and cell growth were observed. Primed UCMSCs expanded *in vitro* formed densely packed colonies with irregular shape and mesh-like structures, whereas the control cell cultures appeared fibroblast-like and more randomly distributed (Figure 1A).

***Colony-forming unit fibroblast activity of primed UCMSCs***

When the cells were seeded at low density, cultured for two weeks, and stained with crystal violet, formed cell colonies were macroscopically clearly visible (Figure 1B). Results from all six CFU-F assays showed that there is a clear tendency for each experiment that IFNγ treated UCMSCs possess a stronger potential to form colonies than the control untreated cells (Figure 1C). Results from the clonogenicity assay showed that when the cell colonies (larger than 50 cells) were counted for each cell line and presented as mean±SD, control cells form significantly fewer colonies compared with primed UCMSCs cells (10.2 ± 8.0 versus 26.0 ± 12.2, respectively; *P*<0.05) (Figure 1D). 

***Immunophenotypic profile of UCMSCs***

**Figure 2:**
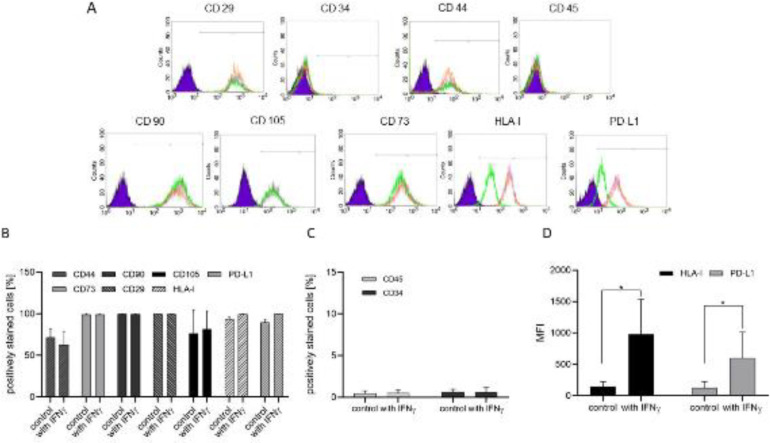
Immunophenotypic characterization of human UCMSCs primed with IFNγ at passage two

Culture-expanded control UCMSCs from all donors (*n* = 8) strongly expressed the specific panel of UCMSCs surface markers (presented as mean ± SD values of the percentage of positively stained cells): CD29 (99.6 ± 0.05), CD44 (71.5 ± 10.14), CD73 (99.0 ± 0.99), CD90 (99.6 ± 0.32) and CD105 (93.5 ± 2.60) (Figures 2A, B), and were negative for the haematopoietic markers CD45 (0.44 ± 0.34) and CD34 (0.63 ± 0.33) (Figures 2 A, C). Untreated UCMSCs were also positive for HLA-I (93.52 ± 2.60) and programmed death ligand 1 (PD-L1, 89.53 ± 3.41) (Figures 2 A, B). On priming the cells for five days with the inflammatory cytokine IFNγ, no significant difference (*p= ns*) of the percentage of positively stained cells was found regarding all of the markers mentioned above: CD29 (99.5 ± 0.20), CD44 (63.0 ± 15.16), CD73 (98.9 ± 0.80), CD90 (99.6 ± 0.25), CD105 (81.7 ± 21.77), CD45 (0.49 ± 0.42), CD34 (0.61 ± 0.59), HLA-I (99.47 ± 0.32), and PD-L1 (99.7 ± 0.14) (Figures 2 B, C). The median fluorescence intensity (MFI) was also explored and it was found that IFNγ treated UCMSCs possess significantly higher MFI levels regarding the expression of HLA-I (980.43 ± 556.64) and PD-L1 (598.04 ± 416.90) compared with MFI for the same markers of untreated control cells – HLA-I (144.97 ± 78.5) and PD-L1 (122.05 ± 103.83) (*P*<0.05) (Figure 2D).

***Proliferation activity***

**Figure 3:**
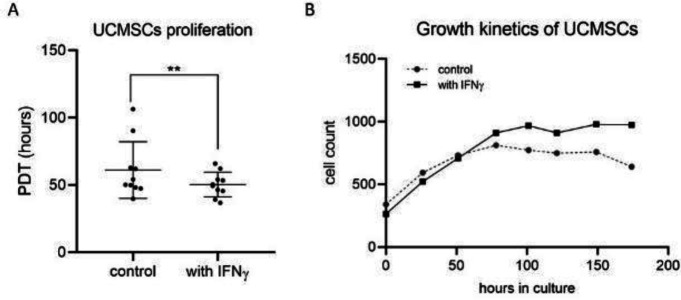
Proliferation activity of UCMSCs primed with the inflammatory cytokine IFNγ

The population doubling time (PDT) of ten different cell lines was calculated and it was estimated that UCMSCs cultured in the presence of IFNγ have a lower PDT compared with the control cultures (50.345 ± 9.155 versus 61.135 ± 21.110, respectively; *P*<0.01) (Figure 3A). As demonstrated by the representative growth curve (Figure 3B) cells enter into the log phase very quickly and grow exponentially until the ~78 hr when they reach the stationary phase, with the IFNγ treated cells reaching higher concentrations. 

***Osteogenic differentiation***

**Figure 4:**
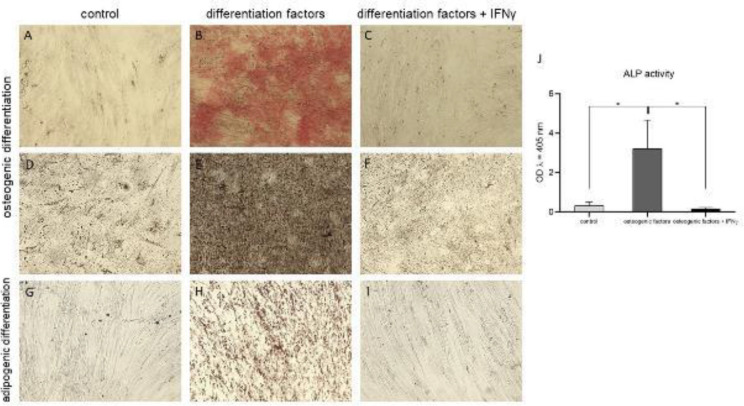
Effect of IFNγ on the differentiation potential of UCMSCs

Cells cultured in the presence of IFNγ (*n*=8) were not able to differentiate osteogenically *in vitro* and did not stain positive with Alizarin red and Von Kossa (Figures 4 C, F). In control cells, treated only with differentiation factors, calcium accumulation was successfully depicted (Figures 4 B, E). This result was further proven by the fact that the alkaline phosphatase activity of cells treated with IFNγ was 19.9-fold lower (0.161 ± 0.06) than that of the cells cultured in medium supplemented only with osteogenic factors (positive control) (3.209 ± 1.44) (*P*<0.05) and was measured close to the negative control cells cultured in plain medium (undifferentiated control cells) (0.314 ± 0.18) (Figure 4J).

***Adipogenic differentiation***

Successful adipogenic differentiation was confirmed cytochemically (*n*=8) with the accumulation of multiple lipid-filled drops in the cytoplasm of a significant percentage of the cells. Cells treated with IFNγ and adipo-inducing factors did not present with an adipogenic phenotype and did not stain positive for triglyceride-containing vacuoles (Figure 4I), and were displaying the same look as the undifferentiated control cells (Figure 4G). Positive control cells (cultured in the presence of adipogenic differentiation factors) presented with multiple lipid vacuoles that stained in red (Figure 4H).

***Cell apoptosis assay***

**Figure 5:**
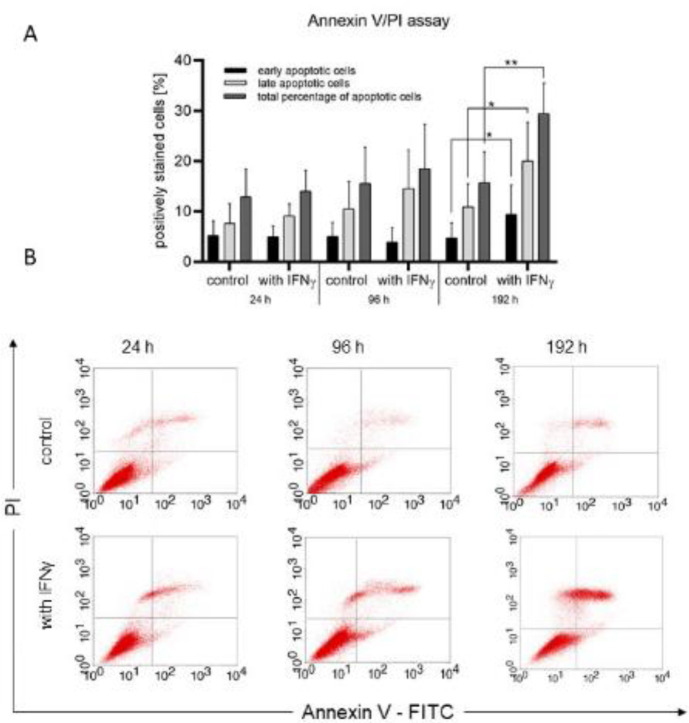
Annexin V/PI flow cytometry assay evaluating the percentage of UCMSCs undergoing early, late, and total apoptosis when exposed to IFNγ

The rate of cells undergoing apoptosis was examined for eight days (*n* = 8). The percentage of cells in early apoptosis (FITC Annexin V positive and PI negative), late apoptosis (FITC Annexin V positive and PI positive), and the total percentage of cells undergoing apoptosis were measured. Control cells were kept in a regular cell culture medium while primed cells were treated with IFNγ. It was found that on the eighth day the percentage of UCMSCs that were exposed to IFNγ in early (9.418 ± 5.847) and late apoptosis (20.052 ± 7.702) was higher than the control cells in early (4.782 ± 2.951) and late apoptosis (10.926 ± 4.565) (*P*<0.05) (Figure 5A). The same result was observed regarding the total percentage of apoptotic cells. On day eight the total percentage of apoptotic cells (Annexin V+/ Annexin V+ and PI+) treated with IFNγ was higher compared with non-treated cells (29.470 ± 6.59 versus 15.708 ±6.190, respectively; *P*<0.01) (Figure 5A). No significant difference was found between the control cells and those treated with IFNγ for the first two measurements on 24^th^ and 96^th^ hours. 

(A) Control UCMSCs and treated with IFNγ cell cultures on day five (magnification, 50x). The primed cells have become more rounded, forming compact colonies. Right panel images represent cell cultures stained with crystal violet. (B) Representative CFU-F assay - control and IFNγ treated cell-derived colonies stained with crystal violet after 14 days of culture *in vitro*. (C) Comparison of CFU-F formation rate between control and IFNγ treated cells for each cell line. (D) Mean ± SD cell colony formation rate (*P<0.05)

CFU-F: colony-forming unit-fibroblasts, IFNγ: Interferon-γ, UCMSCs: umbilical cord mesenchymal stem cells

(A) Representative flow cytometry histograms of the surface markers used to define UCMSCs, HLA-I, and PD-L1 (green – control cultures; red – primed cells). (B-C) UCMSCs cultures (n = 8) were positive for the surface expression of CD44, CD73, CD90, CD29, CD105, HLA-I, and PD-L1, and negative for CD45 and CD34. (D) The median fluorescence intensity (MFI) for HLA-I and PD-L1 of primed and control UCMSCs (*P<0.05). (B-D) Results are presented as mean ± SD

HLA-I: human leukocyte antigen I, IFNγ: Interferon-γ, MFI: median fluorescence intensity, PD-L1: programmed death-ligand 1, UCMSCs: umbilical cord mesenchymal stem cells

(A) The population doubling time (PDT) of control and IFNγ-primed UCMSCs was estimated using Alamar Blue ^®^ assay. (B) Representative growth curve

IFNγ: Interferon-γ, PDT: population doubling time, UCMSCs: umbilical cord mesenchymal stem cells

(A-F) Osteogenic differentiation was confirmed with Alizarin red staining (A-C, A - non-induced control, B – positive control of differentiation, C – cells treated with both osteo-inducing factors and IFNγ), Von Kossa reaction (D-F, D – undifferentiated control cells, E – cells treated with differentiation factors, F - cells cultured with both differentiation factors and IFNγ) and (J) quantitative measurement of ALP activity (results shown as mean ± SD of 8 independent experiments; *P<0.05). (G-I) Oil red O staining of accumulated neutral lipid-filled vacuoles in cells (G - control undifferentiated cells, H – positive control of differentiation, I – IFNγ-primed cells treated with adipogenic factors); magnification, 100x.

ALP: alkaline phosphatase, IFNγ: Interferon-γ, OD: optical density, UCMSCs: umbilical cord mesenchymal stem cells

(A) Percentage of positively stained cells estimated on three different time points. Results are presented as mean ± SD of 8 independent experiments; (*P<0.05; **P<0.01). (B) Representative histograms of the percentage of apoptotic cells

IFNγ: Interferon-γ, PI - propidium iodide

## Discussion

There are numerous studies on the immunomodulatory effects of primed MSCs, but very little information exists on how inflammation affects their characteristic properties (19). IFN-γ is a cytokine that is mainly produced by immune system cells of the innate and adaptive immune responses. Signalization is through the IFN-γ receptor, which has two subunits (IFN-γR1 or CD119 and IFN-γR2), which are expressed on most if not all cell types (20). Upon priming the UCMSCs with IFNγ we observed visible changes in the morphology of the treated cells. When other authors used MSCs from bone marrow (BMMSCs) and adipose tissue (ATMSCs) and treated them with pro-inflammatory cytokines such as IFNγ and TNFα, changes in their morphology were described as well although other authors did not find any transformations (14, 21). In contrast, cells from Wharton’s jelly (WJMSCs) did not show any morphological changes when primed with the pro-inflammatory cytokines (14). Changes in the cell growth kinetics of the primed UCMSCs were also observed. Our results showed that primed UCMSCs have a lower PDT compared with non-primed cells, reaching higher cell concentrations in seven days. These findings corresponded to our data regarding the colony-formation rate of UCMSCs under inflammatory conditions – not only did cells grow faster, but they had higher numbers of CFU-F than control cultures. Our research into the existing literature found that inflammatory conditions lowered the proliferation rate of ATMSCs (22). Primed BMMSCs had weaker proliferative potential than the unprimed cells, but despite the observed changes, none of the cell types lost their ability to differentiate into osteoblasts, chondroblasts, and adipocytes (14, 23). The authors found out that inhibition of the proliferation of mouse and human BMSCs after treating them with IFNγ was through activation of the kynurenine pathway and the subsequent depletion of tryptophan (23). However, treatment of dental pulp MSCs with IFNγ significantly reduced their differentiation potential but their proliferative potential was not affected (24). We, on the other hand, demonstrated that IFNγ primed UCMSCs were not able to differentiate osteogenically and adipogenically. Croitoru-Lamoury *et al*. reported that IFNγ inhibited the gene expression of adipocytic and osteocytic markers in human and mouse BMMSCs (23). Liu *et al*. also reported that proinflammatory T cells inhibited the ability of exogenously added BMMSCs to mediate bone repair, although a relatively high concentration of IFNγ was required to inhibit the osteogenesis of BMMSCs (25). 

We observed that the increased proliferation rate also correlated with a higher percentage of cells in early and late apoptosis on day 8 of culturing the UCMSCs in the presence of IFNγ. In contrast, Liu *et al*. reported that IFNγ synergistically enhanced TNF-α induced MSCs apoptosis, but IFNγ treatment alone did not induce BMMSCs apoptosis (25).

A well-known fact is that the tissue of origin affects the features of MSCs. This is especially normal for the reproductive tissues, where during pregnancy the MSCs are subjected to an enormous variety of cytokines, growth factors, and hormones produced by the many different immune cells found there (26). 

Regarding the expression of surface markers, our results showed that the expression (both percentage positive cells and MFI levels) of the typical MSCs markers (CD29, CD44, CD105, CD73, and CD90) stayed unchanged after treating the cells with IFNγ. Other authors documented the same observations, but only for some of these markers after they kept ATMSCs under inflammatory conditions and tested the expression of CD90, CD105, and CD166 (22). IFNγ also increased the expression of MHC class I and triggered the expression of MHC class II molecules, suggesting that the antigen-presenting property of MSCs can occur (15, 22, 27). 

Emerging evidence leads to the conclusion that the immunosuppressive properties of MSCs are not constitutively turned on. Instead, the surrounding microenvironment, where different inflammatory factors (inflammatory cytokines, like IFNγ, TNFα, and IL-1) are being released from immune cells, licenses the MSCs to acquire immunosuppressive phenotype (28-30). Krampera *et al*. reported, for the first time, the role of IFNγ for the immunosuppressive functions of MSCs (31). The presence of IFNγ is crucial for up-regulation of the expression of the immunological checkpoint molecule programmed death-ligand 1 (PD-L1) from MSCs, so it can bind to the programmed cell death protein 1 (PD-1) on T cells and they can deliver a negative regulatory signal and inhibit T cell proliferation and function (32). Researchers demonstrated the role of PD-L1 for the maintenance, development, and functioning of inducible T regulatory cells (iTregs) (33). Strauch *et al*. reported that applying IFNγ and TNFα on BMMSCs significantly enhanced PD-L1 gene expression, cell surface density, and secretion, which was accompanied by enhanced N-glycosylation (required for PD-L1 to be transported to the cell surface and to be secreted) (34). Our results coincided with the observations of Strauch *et al.* - the surface density expression of PD-L1 on UCMSCs increased significantly when cells were licensed with IFNγ, once again leading to the suggestion that the inflammatory microenvironment is a key regulator for MSCs immunosuppressive role. 

MSCs hold great promise for the treatment of many diseases. They can be recruited to sites of inflammation which brings new hope for therapies using cell-based delivery vesicles (35-37). Exposure to IFNγ induced the expression of chemokine receptors (CCR1, CCR3, CXCR4, CCR5, and CCR10) on MSCs and it also led to a better homing potential towards the sites of inflammation and greater regenerative potential (38). Primed BMMSCs selectively induced the death of tumor cells and showed greater migration potential than untreated cells to sites of inflammation (39, 40). Infusion of BMMSCs and UCMSCs primed with IFNγ reduced the symptoms of Graft-versus-host disease in NOD-SCID mice (41).

Our results and those of previous studies suggest that although MSCs from different tissues have more or less similar surface marker expression profiles, morphology, differentiation potential, and immunomodulatory properties, they can be affected and react differently to the process of inflammation. 

## Conclusion

Based on our results we could hypothesize that under inflammatory conditions UCMSCs transit from their normal resting nature to a state in which they can better fulfill their immunosuppressive functions – possessing stronger proliferative potential, unable to differentiate and express higher levels of PD-L1. These findings could help scientists gain a better understanding of the transformations of MSCs under inflammatory conditions and move forward to designing more effective disease therapies based on stem cells. 

## Authors’ Contributions

KB, IB and DK Study conception and design; KB, IB and EIT Data analyzing and draft manuscript preparation; SK and DK Critical revision of the paper; DK Supervision of the research; KB, IB, EIT, SK and DK Final approval of the version to be published.

## Conflicts of Interest

The authors declare that they have no conflicts of interest.
